# 4-(4-Carboxy­benz­yl)-1-methyl­piperazin-1-ium picrate

**DOI:** 10.1107/S1600536809004474

**Published:** 2009-02-13

**Authors:** Hongqi Li, Q. N. M. Hakim Al-arique, H. S. Yathirajan, B. Narayana, A. R. Ramesha

**Affiliations:** a Key Laboratory of Science and Technology of Eco-Textiles, Ministry of Education, College of Chemistry, Chemical Engineering and Biotechnology, Donghua University, Shanghai 201620, People’s Republic of China; b Department of Studies in Chemistry, University of Mysore, Manasagangotri, Mysore 570 006, India; c Department of Studies in Chemistry, Mangalore University, Mangalagangotri 574 199, India; d RL Fine Chem, Bangalore 560 064, India

## Abstract

The title compound, C_13_H_19_N_2_O_2_^+^·C_6_H_2_N_3_O_7_^−^, is a salt obtained by cocrystallization of 4-[(4-methyl­piperazin-1-yl)meth­yl]benzoic acid and picric acid. The cations adopt an ‘L-shaped’ conformation and are linked into chains along [010] by O—H⋯N hydrogen bonds. The NH group of each piperazinium ring forms a hydrogen bond to the phenolate O atom of a picrate anion, and the picrate anions form face-to-face contacts with an inter­planar separation of 3.023 (1) Å.

## Related literature

For general background, see: Druker *et al.* (2001 ▶). For related structures, see: Swamy *et al.* (2007 ▶); Bindya *et al.* (2007 ▶); Sarojini *et al.* (2007 ▶); Wang & Jia (2008 ▶).
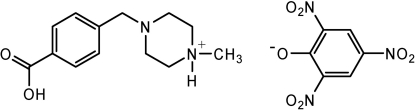


## Experimental

### 

#### Crystal data

C_13_H_19_N_2_O_2_^+^·C_6_H_2_N_3_O_7_^−^
*M* *_r_* = 463.41
Triclinic, 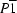

*a* = 7.3020 (12) Å
*b* = 9.5993 (16) Å
*c* = 15.131 (3) Å
α = 86.448 (2)°
β = 79.145 (2)°
γ = 79.950 (2)°
*V* = 1025.2 (3) Å^3^
*Z* = 2
Mo *K*α radiation
μ = 0.12 mm^−1^
*T* = 296 K
0.30 × 0.20 × 0.20 mm

#### Data collection

Bruker SMART CCD diffractometer
Absorption correction: multi-scan (*SADABS*; Sheldrick, 2004 ▶) *T*_min_ = 0.965, *T*_max_ = 0.976
5373 measured reflections
3565 independent reflections
2745 reflections with *I* > 2σ(*I*)
*R*_int_ = 0.016

#### Refinement

*R*[*F* ^2^ > 2σ(*F* ^2^)] = 0.040
*wR*(*F* ^2^) = 0.104
*S* = 1.06
3565 reflections
305 parameters
H atoms treated by a mixture of independent and constrained refinement
Δρ_max_ = 0.18 e Å^−3^
Δρ_min_ = −0.17 e Å^−3^

### 

Data collection: *SMART* (Bruker, 2001 ▶); cell refinement: *SAINT* (Bruker, 2001 ▶); data reduction: *SAINT*; program(s) used to solve structure: *SHELXS97* (Sheldrick, 2008 ▶); program(s) used to refine structure: *SHELXL97* (Sheldrick, 2008 ▶); molecular graphics: *SHELXTL* (Sheldrick, 2008 ▶); software used to prepare material for publication: *SHELXTL*.

## Supplementary Material

Crystal structure: contains datablocks global, I. DOI: 10.1107/S1600536809004474/bi2341sup1.cif

Structure factors: contains datablocks I. DOI: 10.1107/S1600536809004474/bi2341Isup2.hkl

Additional supplementary materials:  crystallographic information; 3D view; checkCIF report

## Figures and Tables

**Table 1:** Hydrogen-bond geometry (Å, °)

*D*—H⋯*A*	*D*—H	H⋯*A*	*D*⋯*A*	*D*—H⋯*A*
O8—H8⋯N4^i^	0.82	1.79	2.6006 (19)	172
N5—H5*A*⋯O1^ii^	0.89 (2)	1.89 (2)	2.734 (2)	156.9 (18)

Symmetry codes: (i) 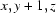; (ii) 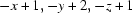.

**Fig. 1.**
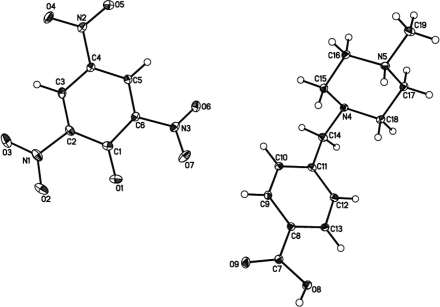
Molecular structure with displacement ellipsoids drawn at the 30% probability level for non-H atoms.

## supplementary crystallographic information

### Comment

4-[(4-Methylpiperazin-1-yl)methyl]benzoic acid is an intermediate in the synthesis of Imatinib, a drug used to treat certain types of cancer. Its mesylate salt is currently marketed by Novartis as Gleevec (Druker *et al.*, 2001). Picric acid forms salts or charge-transfer complexes with many organic compounds. Crystal structures of picrate complexes with organic compounds of pharmaceutical importance *viz.*, desipraminium picrate (Swamy *et al.*, 2007) and amitriptylinium picrate (Bindya *et al.*, 2007) have been reported. A three-dimensional network in piperazine-1,4-diium-picrate-piperazine (1/2/1) is reported recently (Wang & Jia, 2008).

### Experimental

The title compound was prepared by taking equimolar quantities of picric acid (0.92 g, 2 mmol) and 4-[(4-methylpiperazin-1-yl)methyl]benzoic acid (0.47 g, 2 mmol) and dissolving them in water. The solution was stirred well at room temperature and slow evaporation of the solution resulted in the formation of the yellow coloured salt (yield 95%). Crystals suitable for single-crystal X-ray diffraction were grown from dimethyl formamide solvent.

### Refinement

H atoms bound to C atoms were placed at calculated positions and refined using a riding model, with C—H = 0.93–0.97 Å and with *U*_iso_(H) = 1.2*U*_eq_(C) or 1.5*U*_eq_(C) for methyl H atoms. The H atom of the OH group was also placed geometrically and allowed to ride with *U*_iso_(H) = 1.5*U*_eq_(O). The H atom of the NH group was located in a difference Fourier map and refined isotropically without restraint.

### Crystal data

**Table:**

C_13_H_19_N_2_O_2_^+^·C_6_H_2_N_3_O_7_^−^	*Z* = 2
*M**_r_* = 463.41	*F*(000) = 484
Triclinic, *P*1	*D*_x_ = 1.501 Mg m^−^^3^
Hall symbol: -P 1	Melting point = 510–504 K
*a* = 7.3020 (12) Å	Mo *K*α radiation, λ = 0.71073 Å
*b* = 9.5993 (16) Å	Cell parameters from 1962 reflections
*c* = 15.131 (3) Å	θ = 2.5–26.9°
α = 86.448 (2)°	µ = 0.12 mm^−^^1^
β = 79.145 (2)°	*T* = 296 K
γ = 79.950 (2)°	Block, colourless
*V* = 1025.2 (3) Å^3^	0.30 × 0.20 × 0.20 mm

### Data collection

**Table:**

Bruker SMART CCD diffractometer	3565 independent reflections
Radiation source: fine-focus sealed tube	2745 reflections with *I* > 2σ(*I*)
graphite	*R*_int_ = 0.016
φ and ω scans	θ_max_ = 25.0°, θ_min_ = 2.2°
Absorption correction: multi-scan (*SADABS*; Sheldrick, 2004)	*h* = −8→6
*T*_min_ = 0.965, *T*_max_ = 0.976	*k* = −11→9
5373 measured reflections	*l* = −18→18

### Refinement

**Table:**

Refinement on *F*^2^	Secondary atom site location: difference Fourier map
Least-squares matrix: full	Hydrogen site location: inferred from neighbouring sites
*R*[*F*^2^ > 2σ(*F*^2^)] = 0.040	H atoms treated by a mixture of independent and constrained refinement
*wR*(*F*^2^) = 0.104	*w* = 1/[σ^2^(*F*_o_^2^) + (0.0447*P*)^2^ + 0.2218*P*] where *P* = (*F*_o_^2^ + 2*F*_c_^2^)/3
*S* = 1.06	(Δ/σ)_max_ < 0.001
3565 reflections	Δρ_max_ = 0.18 e Å^−^^3^
305 parameters	Δρ_min_ = −0.17 e Å^−^^3^
0 restraints	Extinction correction: *SHELXL97* (Sheldrick, 2008), Fc^*^=kFc[1+0.001xFc^2^λ^3^/sin(2θ)]^-1/4^
Primary atom site location: structure-invariant direct methods	Extinction coefficient: 0.023 (2)

### Special details

**Table:**

Geometry. All e.s.d.'s (except the e.s.d. in the dihedral angle between two l.s. planes) are estimated using the full covariance matrix. The cell e.s.d.'s are taken into account individually in the estimation of e.s.d.'s in distances, angles and torsion angles; correlations between e.s.d.'s in cell parameters are only used when they are defined by crystal symmetry. An approximate (isotropic) treatment of cell e.s.d.'s is used for estimating e.s.d.'s involving l.s. planes.
Refinement. Refinement of *F*^2^ against ALL reflections. The weighted *R*-factor *wR* and goodness of fit *S* are based on *F*^2^, conventional *R*-factors *R* are based on *F*, with *F* set to zero for negative *F*^2^. The threshold expression of *F*^2^ > σ(*F*^2^) is used only for calculating *R*-factors(gt) *etc*. and is not relevant to the choice of reflections for refinement. *R*-factors based on *F*^2^ are statistically about twice as large as those based on *F*, and *R*-factors based on ALL data will be even larger.

### Fractional atomic coordinates and isotropic or equivalent isotropic displacement parameters (Å^2^)

**Table:**

	*x*	*y*	*z*	*U*_iso_*/*U*_eq_	
C1	0.5333 (3)	1.0778 (2)	0.14228 (14)	0.0426 (5)	
C2	0.6431 (3)	1.0691 (2)	0.05175 (13)	0.0438 (5)	
C3	0.7751 (3)	0.9547 (2)	0.02127 (13)	0.0430 (5)	
H3	0.8395	0.9546	−0.0379	0.052*	
C4	0.8126 (3)	0.8398 (2)	0.07793 (12)	0.0379 (4)	
C5	0.7175 (2)	0.83776 (19)	0.16598 (12)	0.0359 (4)	
H5	0.7422	0.7595	0.2039	0.043*	
C6	0.5870 (3)	0.9521 (2)	0.19627 (12)	0.0372 (4)	
C7	0.1636 (3)	1.33630 (19)	0.60024 (12)	0.0366 (4)	
C8	0.1280 (2)	1.18702 (18)	0.60753 (11)	0.0320 (4)	
C9	0.2178 (3)	1.09572 (19)	0.53938 (12)	0.0380 (4)	
H9	0.2943	1.1292	0.4892	0.046*	
C10	0.1949 (3)	0.95535 (19)	0.54527 (12)	0.0391 (4)	
H10	0.2557	0.8954	0.4989	0.047*	
C11	0.0818 (2)	0.90303 (18)	0.61993 (12)	0.0349 (4)	
C12	−0.0108 (2)	0.99528 (18)	0.68722 (12)	0.0361 (4)	
H12	−0.0897	0.9622	0.7367	0.043*	
C13	0.0123 (2)	1.13606 (18)	0.68187 (12)	0.0348 (4)	
H13	−0.0494	1.1964	0.7279	0.042*	
C14	0.0608 (3)	0.74891 (19)	0.62770 (13)	0.0413 (5)	
H14A	−0.0640	0.7413	0.6616	0.050*	
H14B	0.0677	0.7156	0.5677	0.050*	
C15	0.3984 (3)	0.6541 (2)	0.62183 (12)	0.0399 (5)	
H15A	0.4281	0.7488	0.6208	0.048*	
H15B	0.4063	0.6282	0.5601	0.048*	
C16	0.5410 (3)	0.5522 (2)	0.66450 (12)	0.0415 (5)	
H16A	0.5196	0.4562	0.6598	0.050*	
H16B	0.6674	0.5591	0.6324	0.050*	
C17	0.3292 (3)	0.5816 (2)	0.80965 (12)	0.0402 (5)	
H17A	0.3189	0.6046	0.8721	0.048*	
H17B	0.2985	0.4875	0.8083	0.048*	
C18	0.1914 (3)	0.68674 (19)	0.76667 (12)	0.0375 (4)	
H18A	0.0639	0.6846	0.7991	0.045*	
H18B	0.2190	0.7813	0.7701	0.045*	
C19	0.6653 (3)	0.4834 (2)	0.80485 (15)	0.0598 (6)	
H19A	0.6595	0.5121	0.8651	0.090*	
H19B	0.7903	0.4841	0.7710	0.090*	
H19C	0.6355	0.3896	0.8065	0.090*	
N1	0.6141 (3)	1.1856 (2)	−0.01420 (15)	0.0587 (5)	
N2	0.9524 (2)	0.71935 (19)	0.04516 (11)	0.0471 (4)	
N3	0.4925 (2)	0.9427 (2)	0.28970 (11)	0.0469 (4)	
N4	0.2038 (2)	0.65332 (14)	0.67160 (9)	0.0340 (4)	
N5	0.5264 (2)	0.58329 (17)	0.76126 (10)	0.0375 (4)	
O1	0.4059 (2)	1.17657 (15)	0.17135 (11)	0.0614 (4)	
O2	0.5114 (3)	1.2956 (2)	0.00975 (14)	0.0921 (6)	
O3	0.6952 (3)	1.16659 (19)	−0.09220 (12)	0.0799 (6)	
O4	1.0379 (2)	0.72647 (18)	−0.03240 (10)	0.0680 (5)	
O5	0.9804 (2)	0.61680 (16)	0.09632 (11)	0.0649 (5)	
O6	0.4639 (2)	0.82634 (18)	0.32167 (10)	0.0633 (4)	
O7	0.4470 (2)	1.05126 (19)	0.33248 (10)	0.0707 (5)	
O8	0.1067 (2)	1.40458 (13)	0.67570 (8)	0.0445 (4)	
H8	0.1397	1.4825	0.6691	0.067*	
O9	0.2400 (2)	1.38891 (15)	0.53152 (9)	0.0605 (4)	
H5A	0.551 (3)	0.669 (2)	0.7674 (13)	0.045 (6)*	

### Atomic displacement parameters (Å^2^)

**Table:**

	*U*^11^	*U*^22^	*U*^33^	*U*^12^	*U*^13^	*U*^23^
C1	0.0386 (11)	0.0359 (11)	0.0586 (13)	−0.0106 (8)	−0.0166 (9)	−0.0054 (9)
C2	0.0462 (12)	0.0407 (11)	0.0508 (12)	−0.0163 (9)	−0.0195 (10)	0.0092 (9)
C3	0.0449 (11)	0.0537 (13)	0.0350 (10)	−0.0208 (10)	−0.0079 (9)	0.0021 (9)
C4	0.0359 (10)	0.0421 (11)	0.0355 (10)	−0.0087 (8)	−0.0032 (8)	−0.0036 (8)
C5	0.0377 (10)	0.0382 (10)	0.0345 (10)	−0.0097 (8)	−0.0102 (8)	−0.0007 (8)
C6	0.0368 (10)	0.0426 (11)	0.0348 (10)	−0.0100 (8)	−0.0079 (8)	−0.0059 (8)
C7	0.0393 (10)	0.0345 (10)	0.0353 (10)	−0.0079 (8)	−0.0036 (8)	−0.0002 (8)
C8	0.0339 (9)	0.0298 (9)	0.0334 (9)	−0.0060 (7)	−0.0086 (8)	−0.0001 (7)
C9	0.0457 (11)	0.0366 (10)	0.0307 (9)	−0.0071 (8)	−0.0045 (8)	0.0000 (8)
C10	0.0491 (11)	0.0327 (10)	0.0349 (10)	−0.0028 (8)	−0.0081 (9)	−0.0067 (8)
C11	0.0360 (10)	0.0296 (10)	0.0423 (10)	−0.0070 (7)	−0.0141 (8)	0.0004 (8)
C12	0.0311 (10)	0.0339 (10)	0.0415 (10)	−0.0064 (7)	−0.0026 (8)	0.0042 (8)
C13	0.0327 (9)	0.0311 (10)	0.0378 (10)	−0.0029 (7)	−0.0008 (8)	−0.0027 (8)
C14	0.0461 (11)	0.0317 (10)	0.0503 (11)	−0.0104 (8)	−0.0157 (9)	−0.0015 (8)
C15	0.0448 (11)	0.0371 (10)	0.0347 (10)	−0.0087 (8)	0.0013 (8)	0.0020 (8)
C16	0.0423 (11)	0.0371 (11)	0.0385 (10)	−0.0016 (8)	0.0047 (8)	−0.0018 (8)
C17	0.0392 (10)	0.0418 (11)	0.0348 (10)	−0.0040 (8)	0.0030 (8)	−0.0016 (8)
C18	0.0349 (10)	0.0371 (10)	0.0380 (10)	−0.0026 (8)	−0.0013 (8)	−0.0070 (8)
C19	0.0513 (13)	0.0579 (14)	0.0596 (14)	0.0144 (11)	−0.0087 (11)	0.0092 (11)
N1	0.0678 (13)	0.0507 (12)	0.0688 (14)	−0.0265 (10)	−0.0316 (11)	0.0184 (10)
N2	0.0420 (10)	0.0529 (11)	0.0439 (10)	−0.0091 (8)	0.0012 (8)	−0.0076 (8)
N3	0.0368 (9)	0.0614 (12)	0.0420 (9)	−0.0061 (8)	−0.0046 (7)	−0.0111 (9)
N4	0.0385 (8)	0.0289 (8)	0.0338 (8)	−0.0064 (6)	−0.0031 (6)	−0.0028 (6)
N5	0.0363 (9)	0.0317 (9)	0.0407 (9)	−0.0006 (7)	−0.0023 (7)	−0.0001 (7)
O1	0.0513 (9)	0.0413 (9)	0.0904 (12)	−0.0006 (7)	−0.0138 (8)	−0.0095 (8)
O2	0.1085 (16)	0.0526 (11)	0.1104 (16)	0.0045 (11)	−0.0328 (12)	0.0230 (11)
O3	0.1177 (16)	0.0729 (12)	0.0587 (11)	−0.0338 (11)	−0.0321 (11)	0.0242 (9)
O4	0.0656 (10)	0.0825 (12)	0.0461 (9)	−0.0069 (9)	0.0138 (8)	−0.0138 (8)
O5	0.0638 (10)	0.0524 (10)	0.0677 (10)	0.0059 (8)	−0.0006 (8)	0.0038 (8)
O6	0.0623 (10)	0.0701 (11)	0.0503 (9)	−0.0117 (8)	0.0055 (7)	0.0075 (8)
O7	0.0693 (11)	0.0822 (12)	0.0591 (10)	−0.0126 (9)	0.0035 (8)	−0.0371 (9)
O8	0.0599 (9)	0.0308 (7)	0.0409 (7)	−0.0161 (6)	0.0051 (6)	−0.0049 (6)
O9	0.0948 (12)	0.0441 (9)	0.0388 (8)	−0.0271 (8)	0.0105 (8)	0.0014 (6)

### Geometric parameters (Å, °)

**Table:**

C1—O1	1.244 (2)	C14—H14B	0.970
C1—C2	1.450 (3)	C15—N4	1.479 (2)
C1—C6	1.453 (3)	C15—C16	1.509 (3)
C2—C3	1.369 (3)	C15—H15A	0.970
C2—N1	1.466 (3)	C15—H15B	0.970
C3—C4	1.375 (3)	C16—N5	1.493 (2)
C3—H3	0.930	C16—H16A	0.970
C4—C5	1.382 (2)	C16—H16B	0.970
C4—N2	1.447 (2)	C17—N5	1.490 (2)
C5—C6	1.362 (2)	C17—C18	1.505 (2)
C5—H5	0.930	C17—H17A	0.970
C6—N3	1.456 (2)	C17—H17B	0.970
C7—O9	1.209 (2)	C18—N4	1.476 (2)
C7—O8	1.313 (2)	C18—H18A	0.970
C7—C8	1.494 (2)	C18—H18B	0.970
C8—C9	1.385 (2)	C19—N5	1.489 (2)
C8—C13	1.391 (2)	C19—H19A	0.960
C9—C10	1.382 (3)	C19—H19B	0.960
C9—H9	0.930	C19—H19C	0.960
C10—C11	1.390 (3)	N1—O2	1.215 (3)
C10—H10	0.930	N1—O3	1.226 (3)
C11—C12	1.386 (2)	N2—O4	1.225 (2)
C11—C14	1.509 (2)	N2—O5	1.227 (2)
C12—C13	1.386 (2)	N3—O7	1.223 (2)
C12—H12	0.930	N3—O6	1.226 (2)
C13—H13	0.930	N5—H5A	0.89 (2)
C14—N4	1.491 (2)	O8—H8	0.820
C14—H14A	0.970		
			
O1—C1—C2	125.88 (19)	C16—C15—H15A	109.3
O1—C1—C6	122.99 (19)	N4—C15—H15B	109.3
C2—C1—C6	111.10 (16)	C16—C15—H15B	109.3
C3—C2—C1	123.83 (18)	H15A—C15—H15B	108.0
C3—C2—N1	115.70 (19)	N5—C16—C15	111.14 (14)
C1—C2—N1	120.46 (19)	N5—C16—H16A	109.4
C2—C3—C4	120.19 (18)	C15—C16—H16A	109.4
C2—C3—H3	119.9	N5—C16—H16B	109.4
C4—C3—H3	119.9	C15—C16—H16B	109.4
C3—C4—C5	120.72 (17)	H16A—C16—H16B	108.0
C3—C4—N2	119.81 (17)	N5—C17—C18	110.62 (15)
C5—C4—N2	119.47 (17)	N5—C17—H17A	109.5
C6—C5—C4	118.97 (17)	C18—C17—H17A	109.5
C6—C5—H5	120.5	N5—C17—H17B	109.5
C4—C5—H5	120.5	C18—C17—H17B	109.5
C5—C6—C1	125.15 (17)	H17A—C17—H17B	108.1
C5—C6—N3	115.99 (17)	N4—C18—C17	110.48 (14)
C1—C6—N3	118.84 (16)	N4—C18—H18A	109.6
O9—C7—O8	123.05 (17)	C17—C18—H18A	109.6
O9—C7—C8	123.12 (16)	N4—C18—H18B	109.6
O8—C7—C8	113.82 (15)	C17—C18—H18B	109.6
C9—C8—C13	119.15 (16)	H18A—C18—H18B	108.1
C9—C8—C7	118.80 (16)	N5—C19—H19A	109.5
C13—C8—C7	122.01 (15)	N5—C19—H19B	109.5
C10—C9—C8	120.65 (17)	H19A—C19—H19B	109.5
C10—C9—H9	119.7	N5—C19—H19C	109.5
C8—C9—H9	119.7	H19A—C19—H19C	109.5
C9—C10—C11	120.59 (16)	H19B—C19—H19C	109.5
C9—C10—H10	119.7	O2—N1—O3	122.7 (2)
C11—C10—H10	119.7	O2—N1—C2	119.6 (2)
C12—C11—C10	118.64 (16)	O3—N1—C2	117.7 (2)
C12—C11—C14	120.67 (16)	O4—N2—O5	123.64 (17)
C10—C11—C14	120.69 (16)	O4—N2—C4	117.71 (17)
C13—C12—C11	121.00 (17)	O5—N2—C4	118.64 (16)
C13—C12—H12	119.5	O7—N3—O6	123.34 (18)
C11—C12—H12	119.5	O7—N3—C6	118.32 (18)
C12—C13—C8	119.95 (16)	O6—N3—C6	118.34 (17)
C12—C13—H13	120.0	C18—N4—C15	110.02 (14)
C8—C13—H13	120.0	C18—N4—C14	112.50 (13)
N4—C14—C11	115.44 (15)	C15—N4—C14	111.92 (14)
N4—C14—H14A	108.4	C17—N5—C19	111.33 (15)
C11—C14—H14A	108.4	C17—N5—C16	109.59 (15)
N4—C14—H14B	108.4	C19—N5—C16	112.03 (15)
C11—C14—H14B	108.4	C17—N5—H5A	106.5 (13)
H14A—C14—H14B	107.5	C19—N5—H5A	105.9 (13)
N4—C15—C16	111.45 (14)	C16—N5—H5A	111.3 (13)
N4—C15—H15A	109.3	C7—O8—H8	109.5
			
O1—C1—C2—C3	−176.28 (19)	C9—C8—C13—C12	0.4 (3)
C6—C1—C2—C3	2.0 (3)	C7—C8—C13—C12	−177.54 (16)
O1—C1—C2—N1	2.5 (3)	C12—C11—C14—N4	−90.9 (2)
C6—C1—C2—N1	−179.22 (16)	C10—C11—C14—N4	88.9 (2)
C1—C2—C3—C4	−1.1 (3)	N4—C15—C16—N5	−55.9 (2)
N1—C2—C3—C4	−179.94 (17)	N5—C17—C18—N4	59.5 (2)
C2—C3—C4—C5	0.3 (3)	C3—C2—N1—O2	−173.7 (2)
C2—C3—C4—N2	−179.94 (17)	C1—C2—N1—O2	7.4 (3)
C3—C4—C5—C6	−0.7 (3)	C3—C2—N1—O3	6.7 (3)
N2—C4—C5—C6	179.59 (16)	C1—C2—N1—O3	−172.20 (18)
C4—C5—C6—C1	1.9 (3)	C3—C4—N2—O4	2.3 (3)
C4—C5—C6—N3	−179.92 (16)	C5—C4—N2—O4	−177.91 (17)
O1—C1—C6—C5	175.93 (18)	C3—C4—N2—O5	−178.37 (18)
C2—C1—C6—C5	−2.4 (3)	C5—C4—N2—O5	1.4 (3)
O1—C1—C6—N3	−2.2 (3)	C5—C6—N3—O7	146.71 (18)
C2—C1—C6—N3	179.41 (16)	C1—C6—N3—O7	−35.0 (2)
O9—C7—C8—C9	13.3 (3)	C5—C6—N3—O6	−32.8 (2)
O8—C7—C8—C9	−165.86 (16)	C1—C6—N3—O6	145.57 (18)
O9—C7—C8—C13	−168.83 (19)	C17—C18—N4—C15	−58.31 (19)
O8—C7—C8—C13	12.1 (2)	C17—C18—N4—C14	176.17 (15)
C13—C8—C9—C10	−0.7 (3)	C16—C15—N4—C18	56.68 (19)
C7—C8—C9—C10	177.30 (16)	C16—C15—N4—C14	−177.48 (15)
C8—C9—C10—C11	−0.3 (3)	C11—C14—N4—C18	64.0 (2)
C9—C10—C11—C12	1.5 (3)	C11—C14—N4—C15	−60.5 (2)
C9—C10—C11—C14	−178.33 (16)	C18—C17—N5—C19	178.02 (17)
C10—C11—C12—C13	−1.8 (3)	C18—C17—N5—C16	−57.47 (19)
C14—C11—C12—C13	178.01 (16)	C15—C16—N5—C17	55.6 (2)
C11—C12—C13—C8	0.9 (3)	C15—C16—N5—C19	179.69 (17)

### Hydrogen-bond geometry (Å, °)

**Table:**

*D*—H···*A*	*D*—H	H···*A*	*D*···*A*	*D*—H···*A*
O8—H8···N4^i^	0.82	1.79	2.6006 (19)	172
N5—H5A···O1^ii^	0.89 (2)	1.89 (2)	2.734 (2)	156.9 (18)

Symmetry codes: (i) *x*, *y*+1, *z*; (ii) −*x*+1, −*y*+2, −*z*+1.
